# Defining multimorbidity in people with HIV – what matters most?

**DOI:** 10.1097/COH.0000000000000778

**Published:** 2023-01-19

**Authors:** Luxsena Sukumaran, Caroline A. Sabin

**Affiliations:** a Institute for Global Health, University College London; b National Institute for Health Research (NIHR) Health Protection Research Unit (HPRU) in Blood-borne and Sexually Transmitted Infections at University College London, London, UK

**Keywords:** HIV, multimorbidity, multimorbidity definition

## Abstract

Although multimorbidity (defined as the coexistence of multiple conditions) presents significant health challenges to people with HIV, there is currently no consensus on how it should be defined among this population. This review aimed to examine the definition of multimorbidity in existing studies among people with HIV (*n* = 22).

**Recent findings:**

Variation in the definition of multimorbidity (in terms of the number and nature of conditions included) across studies among people with HIV was observed, with less than half (45%) reporting a selection criteria for conditions. The number of conditions considered ranged from 4 to 65. Certain conditions (e.g. stroke, myocardial infarction and chronic kidney disease) and risk factors (e.g. hypertension) were more frequently included, while other symptoms (e.g. joint pain, peripheral neuropathy and sleeping problems) and mental health conditions (e.g. anxiety and panic attacks) were rarely included in the definition of multimorbidity.

**Summary:**

The definition of multimorbidity among people with HIV is highly variable, with certain conditions overlooked. We propose recommendations that researchers should consider when defining multimorbidity among this population to not only enable comparisons between studies/settings but also to ensure studies consider a person-centred approach that can accurately capture multimorbidity among people with HIV.

## INTRODUCTION

Multimorbidity is commonly defined as the coexistence of two or more chronic conditions in an individual [1]. The widespread use of, and advances in, antiretroviral therapy (ART) have markedly improved the life expectancy of people with HIV [2,3]. Consequently, this group is developing comorbidities associated with aging, such as cardiovascular disease, osteoporosis and chronic kidney disease, more frequently and/or at an earlier age than people without HIV [4–6]. By 2030, 84% and 28% of Europeans living with HIV are predicted to have ≥1 and ≥3 comorbidities, respectively [7]. This increased burden adds complexity to HIV clinical care and management, requiring multidisciplinary healthcare expertise. Additionally, the provision of multiple medications (including ART) may compound comorbidity risk and/or cause drug-drug interactions that could lead to clinically suboptimal treatment of HIV and other comorbidities. Whilst several guidelines for the management of comorbidities exist for people with HIV, the complexities of managing people with multiple morbidities may not be fully reflected. Consequently, negative outcomes of multimorbidity, including lower health-related quality of life, higher healthcare utilization and costs, polypharmacy-associated toxicity and mortality, may be exacerbated among people with HIV [8–11].

Given these potential detrimental effects, understanding the prevalence, trends and magnitude of multimorbidity among people with HIV is crucial. However, no clear consensus exists on how to define multimorbidity in this population. Previous reviews have examined the variation in the definition of multimorbidity among non-HIV populations [12,13,14^▪^], with recognition that differences in this have resulted in heterogeneous estimates of multimorbidity prevalence and burden. These differences include the number and type of conditions considered and the sources of morbidity data. The comorbidity profile used to define multimorbidity in people with HIV may, however, be more complex than that of non-HIV populations, including a wider (and potentially different) range of comorbidities, that may be of low prevalence in general populations and which may have occurred as a result of HIV infection itself or its treatment [15,16]. Therefore, applying a definition of multimorbidity that was derived in the general population may result in an under-estimate of the true burden of multimorbidity experienced by people living with HIV.

Here, we present a narrative review of the literature on multimorbidity among people with HIV, with a particular focus on how multimorbidity is defined. By providing a comprehensive synthesis of current evidence we aim to identify common themes/gaps, highlight research priorities, and discuss implications for clinical practice. 

**Box 1:**
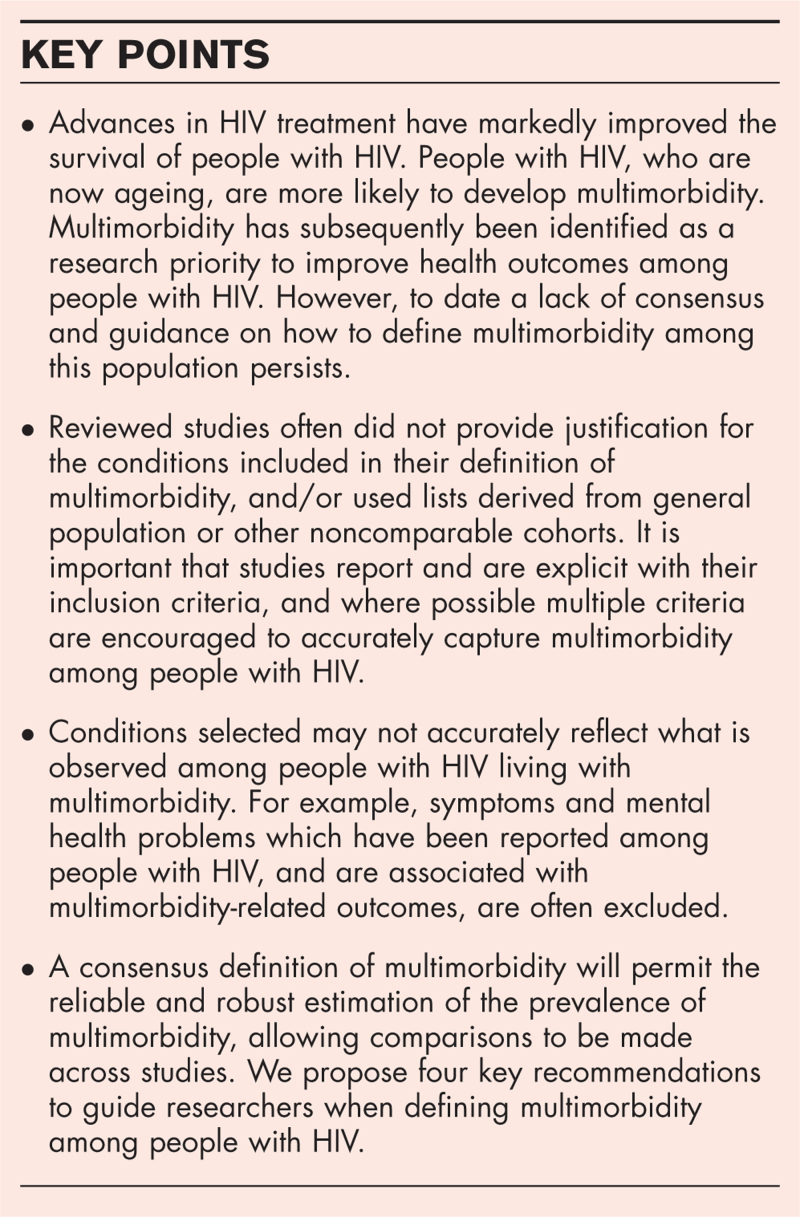
no caption available

## METHODS

We systematically searched the following electronic library databases in September 2022: MEDLINE (Ovid), Web of Science (Clarivate Analytics), and Scopus (Elsevier). Our aim was to identify original research papers that measured multimorbidity in people with HIV. The search was restricted to full-text English-language publications in peer-reviewed journals. Relevant articles were identified using search terms (variations of multimorbidity, measure and HIV, Table S1, Supplemental Digital Content) that were applied to titles in all databases, and we restricted searches to studies in adults aged ≥18 years. This review was conducted in accordance with the Preferred Reporting Items for Systematic Reviews and Meta-Analyses (PRISMA) guidelines and checklist (Table S2, Supplemental Digital Content).

We selected studies that explicitly state the definition and measurement used to examine multimorbidity in populations of people with HIV, and therefore not all study designs were eligible for inclusion. We also restricted this review to studies that stated the conditions included in their definition of multimorbidity to identify the trends and implications of conditions considered in multimorbidity research among people with HIV.

**FIGURE 1:**
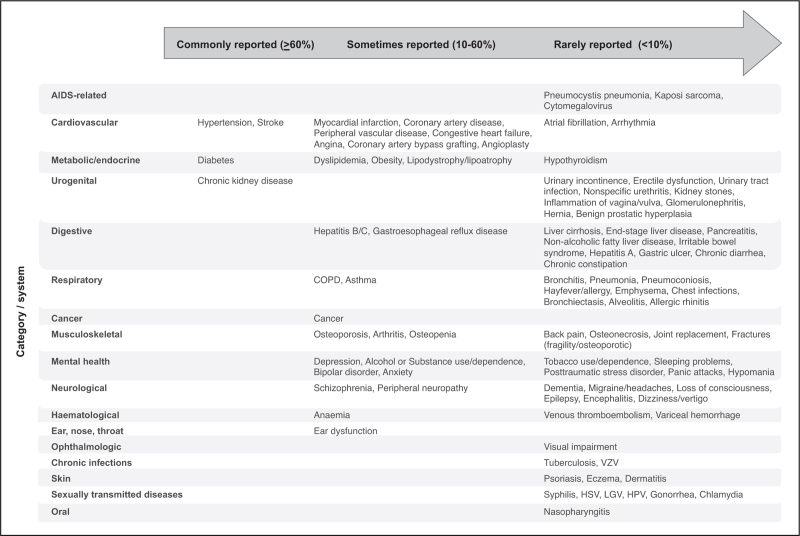
Conditions included in reviewed studies, categorized into commonly (≥60%), sometimes (10–60%) and rarely reported (<10%).

Our initial database search identified 117 articles, which was reduced to 59 after removing duplicates. After abstract review, 25 articles were selected for full text review, of which 22 were included (Figure 1, Supplemental Digital Content).

## RESULTS

### Study characteristics

A majority of studies (63.6%) were published in the last 4 years (Table S3, Supplemental Digital Content). The number of people with HIV included varied from 189 to 38 868 (median 1080, interquartile range [IQR] 651–5368). Most studies were conducted in North America (9 [40.9%]), followed by Europe (7 [31.8%]). Multimorbidity was most commonly examined in community-based settings (12 [54.5%]) (Table 1). Most studies (18 [81.8%]) focused on all adults (those aged ≥18 years), whereas a smaller proportion included middle-aged/older adults (≥45 years) (3 [13.6%]) or older adults (aged ≥65 years) (1 [4.5%]) only. Morbidity data was predominately sourced from electronic health records (17 [77.3%]). Over half of studies (16 [72.7%]) defined multimorbidity as the presence of ≥2 conditions, with other studies defining multimorbidity as the presence of ≥1 condition (2 [9.1%]), or ≥3 conditions (1 [4.5%]); three studies (13.6%) did not state a reference definition. The most common purpose for measuring multimorbidity was to examine potential risk factors (13 [59.1%]), followed by identification of multimorbidity patterns/clusters (6 [27.3%]). Fourteen studies (63.6%) used a simple count of conditions to measure multimorbidity, one study (4.5%) used weighted indices (Cumulative Illness Rating Scale), and two (9.1%) used both types of measures together. Other measures included those derived using statistical approaches (6 [27.3%]) such as cluster, factor or principal component analysis.

**Table 1:** Summary of study characteristics

Study characteristic	No. of studies (*n* = 22)	%
Study setting		
Community	12	54.5
Secondary/tertiary care	7	31.8
Hospital	3	13.6
Data source		
Electronic health records	17	77.3
Self-reported	3	13.6
Self-reported/health records	1	4.5
Administrative data	1	4.5
Study population		
All adults (aged ≥18 years)	18	81.8
Middle-aged and older adults (aged ≥45 years)	3	13.6
Older adults (≥65 years)	1	4.5
Study purpose		
Association of risk factors with multimorbidity	13	59.1
Patterns or clusters of MM	6	27.3
Prevalence or burden of multimorbidity (without examining associations with risk factors or outcomes)	2	9.1
Association of multimorbidity with outcome	1	4.5
Definition for multimorbidity		
≥1	2	9.1
≥2	16	72.7
≥3	1	4.5
Not stated	3	13.6
Type of multimorbidity measure		
Simple count	14	63.6
Weighted index	1	4.5
Simple count & weighted index	1	4.5
Statistical approach	6	27.3
Number of conditions included in measure		
1–10	12	54.5
11–20	6	27.3
21–30	3	13.6
31–40	0	0.0
41–50	0	0.0
>50	1	4.5
How were conditions considered		
Individual	7	31.8
Grouped & individual	15	68.2
Condition selection criteria stated		
Yes	10	45.5
No	12	54.5

### The number of conditions selected

The number of conditions considered in each definition of multimorbidity has varied across studies among people with HIV, ranging from 4 to 65 (median 10, IQR 7–15) (Table S3, Supplemental Digital Content). Over half of studies reviewed included <10 conditions, while other studies included 11–20 (*n* = 6 [27.3%]) and 21–30 conditions (*n* = 3 [13.6%]); only one study included >30 conditions (65 comorbidities were included in the analysis) (Table 1) [17]. Heterogeneity in the number of conditions included is likely to influence prevalence estimates of multimorbidity. Furthermore, while general population studies often refer to a review from Fortin *et al.*[18] when selecting the number of conditions (this review recommends authors to select >12 conditions), none of the studies among people with HIV provided a justification for why the specific number of conditions was chosen.

### Selection criteria for conditions

Less than half of the reviewed studies provided selection criteria for conditions included in their multimorbidity definition. Among studies that stated selection criteria (*n* = 10), five reported one criterion, four reported two and one reported four criteria (Table 2). These selection criteria are discussed below.

**Table 2:** Selection criteria used by reviewed studies (*n* = 10)

Author	No. of selection criteria	Selection criteria category	Selection criteria
Wong *et al.* 2018	4	Prevention, prevalence, clinical significance & review of studies	• Amenable to primary and secondary prevention • Higher occurrence among people with HIV • Contribute to causes of death among people with HIV • Inclusion in other multimorbidity studies among people with HIV
Guaraldi *et al.* 2018	1	Guidelines	• Based on European AIDS Clinical Society (EACS) guidelines (2018)
Arant *et al.* 2021	2	Prevalence & review of studies	• Higher occurrence among people with HIV • Inclusion in other multimorbidity studies (Scouten *et al.* 2014 & Kim *et al.* 2021)
Castilho *et al.* 2019	1	Clinical significance	• Clinical significance (in terms of morbidity and mortality)
Edmiston *et al.* 2015	2	Prevalence & guidelines	• Higher occurrence among people with HIV (Deeks *et al.* 2009 & EACS Guidelines 2013)
Kim *et al.* 2012	2	Clinical significance & review of studies	• Review of multimorbidity literature • Relevance and clinical significance to people with HIV (determined by three of the authors)
Mefford *et al.* 2022	2	Clinical significance & guidelines	• Recommendations by the US Department of Health and Human Services Strategic Framework on Multiple Chronic Conditions and previous work conducted by the Cardiovascular Research Network (CVRN). • Relevant to placing persons at increased risk for hospitalisation
Yang *et al.* 2021	1	Review of studies	• Review of existing comorbidity literature in both the general and HIV-infected populations (Althoff *et al.* 2015; Kim *et al.* 2012; van den Bussche *et al.* 2011)
Ahmed *et al.* 2022	1	Prevalence	• ’Top 30 co-occurring conditions” in the study population
De Francesco *et al.* 2018	1	Prevalence	• Conditions with a prevalence ≥1.5% in the study population

#### Review of multimorbidity literature

Although four of the reviewed studies selected conditions that were included in previous multimorbidity studies, these reference studies often had distinct, noncomparable, study characteristics (e.g. were conducted among on Veterans living with HIV [4], injection drug users [19], or those with elevated body mass index [BMI]) [20]. One study, referenced by the other three, focused on a US-based population (aged ≥19 years) and considered 15 conditions [20], selected based on previous literature in both the general and HIV-populations. Another reference study was conducted within an older (65 years) German population without HIV [21], but was used by a US-based study measuring multimorbidity in people with HIV (aged ≥18 years) [22]. This German study selected the most frequent conditions among attendees at general practitioner (GP) surgeries, restricting their analysis to conditions with a prevalence >1% in those aged >65 years, which included chronic liver disease and dementia. However this list may not accurately capture conditions more commonly seen in younger populations or in those from different geographical regions or healthcare settings. Additionally, selection of conditions based on those observed in GP attendees may not necessarily reflect the conditions with the greatest burden (in terms of healthcare utilization or health outcomes) to an individual or healthcare system. Another US study [23] based their selection on what had been included in a Dutch study [16]. Although both studies considered a similar age group (aged ≥45 years), the reference study based their condition list on the availability of data (clinical/laboratory) rather than on the clinical significance of conditions among people with HIV.

#### Guidelines/recommendations from health organizations

Three studies [24–26] selected their conditions based on guidelines from either the European AIDS Clinical Society (EACS) [27] or the US Department of Health and Human Services (DHHS) [28]. Selection of conditions based on the EACS guidelines suggests that authors have considered relevant conditions for people with HIV. In contrast, the DHHS guidelines offer generalized recommendations for the American population, and thus the comorbidities included may not necessarily reflect those that are most burdensome among people with HIV.

#### Prevalent conditions among people with HIV

Five studies selected conditions based on their prevalence among people with HIV. Two of these stated that they included the ‘top 30 chronic conditions’ and conditions with a prevalence ≥1.5%, but no reasoning was provided for these specific thresholds [17,29]. A 2015 Australian study selected common conditions among people with HIV, referencing EACS guidelines and a review published in 2009 [30]. However, the latter may have provided an outdated representation of relevant conditions among people with HIV at the time of the later study. Additionally, two US studies [23,31] referred to a publication from the AGEhIV Cohort Study in the Netherlands. However, there are likely to be country-level differences in terms of prevalent conditions among people with HIV which may have not been considered.

#### Relevance and clinical significance

Four studies included conditions based on their clinical significance. Specifically, two studies included conditions associated with mortality, one of which used mortality trends from the Data collection on Adverse events of anti-HIV Drugs (D:A:D) study (212 clinics across Europe, USA and Australia) [31], whereas the other did not provide references to support this selection criterion [32]. Similarly, authors from another study stated that their final list of conditions was chosen based on relevance and clinical significance to people with HIV [20]. How this was determined was not described by authors but could potentially be driven by conditions associated with obesity (a central focus of their analysis). Furthermore, a study that examined the association of multimorbidity in people with HIV with incident heart failure selected conditions associated with increased risk for hospitalization among adults with heart failure. However, authors did not consider conditions associated with this outcome among people with HIV generally [25]. It is important that conditions are selected based on clinical significance, but authors should consider conditions that contribute to a broader physical and psychosocial burden (e.g. everyday functioning, quality of life and treatment burden) rather than solely focusing on severe outcomes (e.g. mortality or hospitalization). A holistic approach that considers these conditions will provide a more accurate representation of multimorbidity among this population with which to better inform future care pathways.

### Conditions included in multimorbidity studies among people with HIV

**FIGURE 2:**
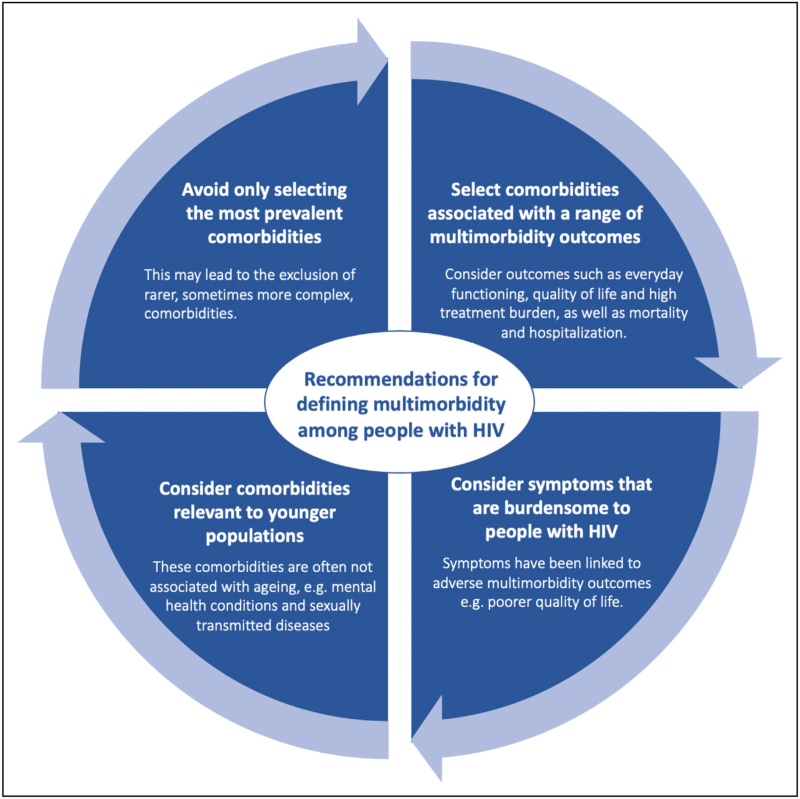
Four key recommendations for researchers when defining multimorbidity among people with HIV.

All studies measuring multimorbidity in people with HIV included a cardiovascular and metabolic condition (Figure 2, Supplemental Digital Content). Over half of the studies also included at least one urogenital, digestive, respiratory, musculoskeletal, and malignancy condition in their list. In contrast, only a small proportion of studies included chronic infections, ophthalmological, ear/nose/throat, and skin disorders. Oral and congenital conditions were not included in any of the reviewed studies. Among cardiovascular/metabolic conditions, diabetes was included by all studies (Fig. 1); hypertension, chronic kidney disease, stroke, chronic obstructive pulmonary disease, dyslipidaemia, and myocardial infarction were also commonly included across studies. In contrast, sexually transmitted diseases (chlamydia, gonorrhoea and human papillomavirus), AIDS-related events (*Pneumocystis* pneumonia, Kaposi's sarcoma and cytomegalovirus) and neurological disorders (migraines/headaches, epilepsy and encephalitis) were included in <5% of studies.

#### Inclusion of mental health conditions

Many studies (55%) only included physical comorbidities, while less than half included both physical and mental health comorbidities. Mental health conditions, including depression and anxiety, are more prevalent among people with HIV compared to those without HIV [33–35]. Factors contributing to an increase in psychological morbidity among those living with HIV include negative experiences and/or stigma around disclosure of HIV status and traumatic losses during the HIV epidemic [36]. Despite this, several studies continue to exclude mental health conditions when examining multimorbidity in this population. Moreover, among those that do incorporate mental health conditions, most only include depression (36%), whereas other mental health conditions such as sleeping problems, panic attacks and anxiety were included in less than 10% of studies. These conditions may also be important in terms of their individual (and synergistic) impact on everyday functioning and quality of life.

#### Defining beyond conditions and risk factors

The majority of reviewed studies often selected chronic conditions and/or risk factors (defined as ‘conditions or measurements associated with the probability of disease or death’ [37]) when defining multimorbidity. However, this may not necessarily capture the priorities of people with HIV with multimorbidity. Inclusion of symptoms (defined as ‘any expression of disturbed function or structure of the body and mind by patient’ [38]) may provide a deeper understanding of multimorbidity from the perspectives of people living with HIV. Although we recognize that symptoms are not necessarily an indication of an underlying disease [37], they may be sequelae of an undiagnosed and untreated condition. Additionally, people with HIV are likely to experience a higher number of symptoms compared to the general population due to contributions from prolonged ART exposure and HIV-mediated persistent inflammation, and therefore their inclusion may be relevant when defining multimorbidity among this specific population. This is of particular relevance as people with HIV have reported that the presence of symptoms may reduce an individual's ability to carry out routine day-to-day tasks and health-related quality of life [39,40^▪▪^,^41^]. Such symptoms include cognitive (e.g., memory problems and sleep disturbance) and physical (e.g. joint pain/neuropathy and gastrointestinal issues) complaints. Despite this, only a small proportion of reviewed studies (36%) included at least one of the following symptoms: angina (23%), back pain (9%), constipation (5%) and diarrhoea (5%).

#### Inclusion of conditions relevant to younger populations

Although people with HIV are more likely to develop age-associated comorbidities, multimorbidity is increasingly reported in younger adults [42,43]. Reviewed studies that included adults aged ≥18 years, however, often included conditions in previous studies conducted among middle-aged (≥45 years) and/or older (≥65 years) populations. These conditions, which are typically prevalent in older populations, included chronic liver and kidney disease, stroke and myocardial infarction. Therefore, conditions that may contribute to multimorbidity among younger populations, including sexually transmitted diseases and mental health conditions, may have been missed in their definition.

## CONCLUSION

In this review, we have highlighted that definitions of multimorbidity vary significantly among studies of people with HIV. Drawbacks of many of the definitions used include the small number of conditions that are considered despite the far wider range of comorbidities generally seen in those with HIV. Conditions are often selected for inclusion based on lists used in previous studies with distinct population characteristics including people without HIV. Thus, conditions of particular relevance to people with HIV, including symptoms and mental health problems, continue to be overshadowed by conditions that are highly prevalent in different populations or that demonstrate a strong association with mortality and/or hospitalisation. We recognize that the definition will be subjective, depending on the research question, and may need to be pragmatic. However it is important that researchers are explicit about the selection criteria used. Based on our findings we also propose recommendations (Fig. 2) that we believe will capture and address the complexities of defining multimorbidity specifically among people with HIV.

## Acknowledgements

*We acknowledge members of the NIHR HPRU Steering Committee: Professor Caroline Sabin (HPRU Director), Dr John Saunders (UKHSA Lead), Professor Catherine Mercer, Dr Hamish Mohammed, Professor Greta Rait, Dr Ruth Simmons, Professor William Rosenberg, Dr Tamyo Mbisa, Professor Rosalind Raine, Dr Sema Mandal, Dr Rosamund Yu, Dr Samreen Ijaz, Dr Fabiana Lorencatto, Dr Rachel Hunter, Dr Kirsty Foster and Dr Mamoona Tahir. The views expressed are those of the authors and not necessarily those of the NIHR, the Department of Health and Social Care, or the UKHSA.*

### Financial support and sponsorship

*LS was funded through the National Institute for Health and Care Research Health Protection Research Unit (NIHR HPRU, Grant no: NIHR200911) in Blood Borne and Sexually Transmitted Infections at University College London in partnership with the UK Health Security Agency (UKHSA). The views expressed are those of the authors and not necessarily those of the NIHR, the Department of Health and Social Care, or the UKHSA.*

### Conflicts of interest

*C.S. has received funding from Gilead Sciences and ViiV Healthcare for membership of Advisory Boards and for preparation of educational materials. L.S. reports no conflicts of interest.*

## Supplementary Material

Supplemental Digital Content

## REFERENCES AND RECOMMENDED READING

Papers of particular interest, published within the annual period of review, have been highlighted as:

▪ of special interest
▪▪ of outstanding interest
